# Propofol and Sevoflurane Differentially Modulate Cortical Depolarization following Electric Stimulation of the Ventrobasal Thalamus

**DOI:** 10.3389/fncom.2017.00109

**Published:** 2017-12-11

**Authors:** Stephan Kratzer, Corinna Mattusch, Paul S. Garcia, Sebastian Schmid, Eberhard Kochs, Gerhard Rammes, Gerhard Schneider, Matthias Kreuzer, Rainer Haseneder

**Affiliations:** ^1^Department of Anesthesiology, Klinikum rechts der Isar, Technische Universität München, Munich, Germany; ^2^Department of Anesthesiology, Emory University, Atlanta, GA, United States; ^3^Research Service, Atlanta VA Medical Center, Atlanta, GA, United States

**Keywords:** propofol, sevoflurane, thalamocortical, unconsciousness, mechanisms of anesthesia

## Abstract

The neuronal mechanisms how anesthetics lead to loss of consciousness are unclear. Thalamocortical interactions are crucially involved in conscious perception; hence the thalamocortical network might be a promising target for anesthetic modulation of neuronal information pertaining to arousal and waking behavior. General anesthetics affect the neurophysiology of the thalamus and the cortex but the exact mechanisms of how anesthetics interfere with processing thalamocortical information remain to be elucidated. Here we investigated the effect of the anesthetic agents sevoflurane and propofol on thalamocortical network activity *in vitro*. We used voltage-sensitive dye imaging techniques to analyze the cortical depolarization in response to stimulation of the thalamic ventrobasal nucleus in brain slices from mice. Exposure to sevoflurane globally decreased cortical depolarization in a dose-dependent manner. Sevoflurane reduced the intensity and extent of cortical depolarization and delayed thalamocortical signal propagation. In contrast, propofol neither affected area nor amplitude of cortical depolarization. However, propofol exposure resulted in regional changes in spatial distribution of maximum fluorescence intensity in deep regions of the cortex. In summary, our experiments revealed substance-specific effects on the thalamocortical network. Functional changes of the neuronal network are known to be pivotally involved in the anesthetic-induced loss of consciousness. Our findings provide further evidence that the mechanisms of anesthetic-mediated loss of consciousness are drug- and pathway-specific.

## Introduction

The underlying mechanisms leading to anesthetic-induced loss of consciousness remain unclear. There is a common agreement that thalamic and cortical areas are major targets of anesthetic substances and that thalamocortical interactions are crucial for maintenance of consciousness (Mashour, 2014). Interfering with information processing between cortex and thalamus may represent a key mechanism of anesthetic-induced unconsciousness. Alkire and colleagues proposed that losing consciousness either occurs by a loss of cortical integration or by a reduction of information capacity (Alkire et al., 2008). This means that either the processing of relevant information is impaired or disrupted during anesthetic-induced unconsciousness, or that the activity patterns representing the information are reduced, insufficient to maintain conscious states. Electrophysiological studies showed that anesthetics decrease the information content in the signal and disrupt or impair directed information flow, i.e., cause an impairment of cortical feedback connectivity and frontal-parietal communication (Ku et al., 2011; Jordan et al., 2013; Ranft et al., 2016). These EEG analyses are restricted to cortical activity, and can only infer the influence of subcortical structures like the thalamus.

Our experiments focused on anesthetic induced changes in cortical neuronal activity following a thalamic electrical stimulus in acute murine slice preparations. Anesthetic substances of different chemical compositions produce a similar phenotypic endpoint—unconsciousness—despite likely differences in their mode of action (Rudolph and Antkowiak, 2004; Franks, 2006). By means of voltage-sensitive dye imaging (VSDI) we intended to evaluate the difference in drug-induced changes to post-stimulus fluorescence between the volatile anesthetic sevoflurane and the intravenous agent propofol. While propofol mainly acts via the GABA_A_ receptor, sevoflurane affects a broader range of molecular targets, such as GABA_A_, glycine or glutamate receptors (Campagna et al., 2003; Rudolph and Antkowiak, 2004). Our VSDI approach with thalamic stimulation allows real-time tracking of stimulus propagation from thalamus to cortex by a fluorescent response caused by potential changes in the neuron's membrane. Therefore, we stained acute murine slice preparations with preserved functional thalamocortical network using a fluorescent dye and evaluated the cortical response in different cortical layers after a voltage stimulus was applied to thalamic structures in the absence and presence of either sevoflurane or propofol. The focus of our investigations was on a possible difference in the anesthetics' effect on thalamocortical signal propagation, based on the different molecular action profiles of sevoflurane and propofol.

## Materials and methods

The described experimental protocols were approved by the Ethical Committee on Animal Care and Use of the Government of Bavaria (Munich, Germany).

### Thalamocortical slice preparation

We removed the brains from 10 male C57Bl6/N mice (P28–P49) under isoflurane anesthesia and prepared one thalamocortical slice per animal that contained the ventrobasal thalamus and the sensorimotor barrel cortex using a vibratome (HM 650 V, Microm International, Walldorf, Germany) as described by Agmon and Connors (Agmon and Connors, 1991) in ice-cold artificial sucrose-based cerebrospinal fluid (aCSF: 2.5 mM KCl, 24 mM NaHCO_3_, 1.25 mM NaH_2_PO_4_, 234 mM Sucrose, 11 mM glucose, 0.5 mM CaCl_2_, 10 mM MgSO_4_; pH 7.4) that was saturated with a carbogen-gas mixture (95% O_2_/5% CO_2_). Each slice was 400 μm thick. We allowed the slices to recover for at least 1 h at 34°C after the preparation. We used standard aCSF [(in mM): NaCl, 125; KCl, 2.5; NaHCO_3_, 25; CaCl_2_, 2; MgCl_2_, 1; D-glucose, 25; NaH_2_PO_4_, 1.25, substances purchased from Sigma-Aldrich (Munich, Germany)] to incubate the slices.

We stained the slices with the dye Di-4-ANEPPS (7.5 mg/ml; <0.1% DMSO) (Stepan et al., 2012) that was added to the carbogen-saturated standard aCSF for 15 min. and allowed the slices to recover for at least 30 min in standard aCSF at room temperature.

DI-4-ANEPPS is a styryl dye, which after rapid internalization in the neuronal cell reacts to changes in the neuronal membrane potential with changes in the fluorescence intensity in the millisecond-range. Di-4-ANEPPS responds to depolarization of the neuronal membrane with a decrease in fluorescence when excited at approximately 530 nm (Fluhler et al., 1985).

We continuously perfused the recording chamber with the standard aCSF at a flow rate of 5–8 ml/min and induced cortical depolarization by application of electrical stimuli of 50–100 V for 0.5 ms to the ventroposteromedial thalamus via a bipolar concentric electrode (SNEX-100, Hugo-Sachs, March, Germany). The stimulation electrode had a tip diameter of 100 μm. Stimulus intensity was adjusted to obtain a sub-maximum (regarding area and amplitude) cortical response.

### Application of the anesthetic substance

We recorded a stable baseline for 30 min with the slice kept only in standard aCSF before we delivered the anesthetic substance sevoflurane (*n* = 5) or propofol (*n* = 5). We added sevoflurane to the perfusate by passing the carbogen-gas mixture through a calibrated agent specific vaporizer (Dräger, Lübeck, Germany) prior to aerating the aCSF with it. The aqueous concentrations of sevoflurane correlate with the applied vapor dial settings in a linear fashion as published previously (Haseneder et al., 2009). We present the concentrations as volume percent (vol%) and refer to the dial settings on the vaporizer. We applied sevoflurane in subsequently increasing concentrations (0.6, 1.6, and 3.2%), where 3.2% sevoflurane corresponds to the published minimum alveolar concentration of sevoflurane in mice (Ichinose et al., 1998). For each concentration of a substance we waited 30 min before recordings to allow equilibration of the substance. We dissolved propofol in dimethyl sulfoxide and administered it directly in stepwise increasing concentrations (0.3, 0.6, 1.0, and 3.0 μm) to the recirculating aCSF. Similar to sevoflurane, we allowed 30 min equilibration before starting the experiments.

### Optical recording

We performed the VSDI with an Olympus BX51WI fluorescence microscope (Olympus, Hamburg, Germany) that was equipped with a MiCAM02-HR camera (BrainVision, Tokyo, Japan) as well as a XLFluor4X/340 objective (NA 0.28; Olympus, Hamburg, Germany) with a 480–550 nm band pass excitation filter, a 590 nm dichroic, and a 590 nm low emission filter. The relative change in recorded fluorescence (Δ*F/F*) of the dye served as correlate of neuronal activity. We recorded *F* in an 88 × 60 pixel frame with 36.4 × 40.0 μm pixel dimensions at a sampling (frame) rate of 2.2 ms. We reduced the pixelation of images with the interpolation function of the MiCAM02 software (BrainVision, Tokyo, Japan) and improved the signal-to-noise ratio by recording and averaging eight stimulation runs in 15 s intervals. After recording we processed the data with functions provided by the BrainVision Software. We spatially smoothed the Δ*F/F* values with a 3 × 3 pixel average filter. Additionally, we applied a temporal filter *F(t)* = *(F(t* − *1)* + *F(t)* + *F(t* + *1))/3* to the pixels' *F(t)*-values, where *t* is the frame number.

For the consequent analyses with MATLAB® R2012a (The MathWorks Inc., Natick, MA, USA), we exported the recorded experiments to a text file with the BrainVision software. For evaluation of the effects of sevoflurane and propofol on the amplitude and area of cortical depolarization the average of eight recordings before application of the anesthetic (baseline) was compared to the averaged value of eight recordings in the presence of the given concentration after equilibration time.

### Analysis of post-stimulus cortical activity

We assessed a set of parameters to evaluate the impact of sevoflurane or propofol on the cortical response of neural activity after a thalamic stimulation.

#### General cortical response

In a first step, we investigated the change of the overall cortical response to the stimulus. The parameters of choice were the overall fluorescent intensity *I*_*total*_ as well as the size of the activated cortical area *A*_*total*_ after the stimulus. A pixel was defined as active if it showed an intensity value higher than the 5-fold standard deviation of the background intensity (noise) at non-stimulated conditions.

For this investigation, we manually selected the cortex as a region of interest (ROI) with the MATLAB® *polygon* function. *A*_*total*_ is the area, i.e., the number of pixels, within the defined ROI that exceeded the five-fold standard deviation of the mean background fluorescence activity of the entire slice prior to stimulation. *I*_*total*_ is the average of the maximum *F*-value of each pixel within a timeframe of 220 ms after stimulation. The 220 ms correspond to 100 frames of the recording. We chose this long observation period to ensure that we capture the entire stimulus response.

#### Response in cortical pixel bands

**Figure 1:**
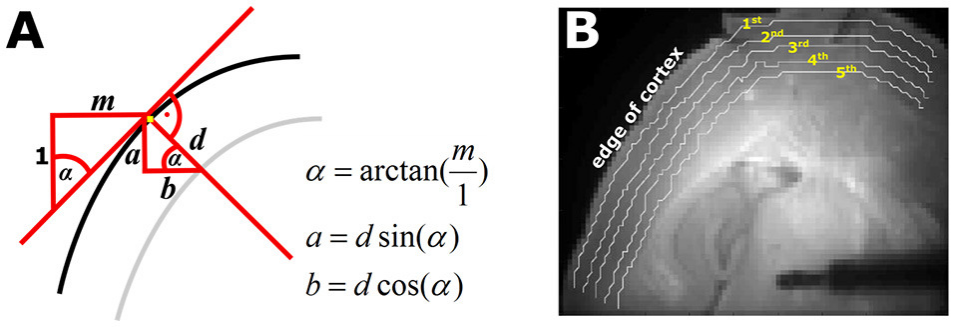
Automated selection of the five pixel bands. **(A)** Geometrical sketch of how the pixel bands were generated. The black line represents the edge of the cortex. The red line indicates the calculated slope *m* of cortical edge at the observed position, depicted by the yellow pixel. With the knowledge of *m* and the defined depth *d* of the pixel band, the parameters *a* (vertical) and *b* (horizontal) necessary to automatically generate the pixel bands can be generated. The gray line is the automatically generated pixel band at depth *d*. **(B)** Picture of a thalamocortical slice as used for the experiments. The white lines indicate the automatically selected 1st−5th pixel bands as depicted by the yellow numbers.

We designed a MATLAB® routine to automatically define five pixel bands with a distance of approximately 100 μm that run parallel to the outer edge of the cortex, after manually defining this cortical edge. We used the selected pixels defining the cortical edge to calculate a 3rd order polynomial fit of the edge with the MATLAB® *polyfit* function. Based on the fitted function of shape *P(x)* = *p*_1_*x*^3^ + *p*_2_*x*^2^ + *p*_3_*x* + *p*_4_ and its first derivate *P*′*(x)* = *3p*_1_*x*^2^ + *2p*_2_*x* + *p*_3._ The 1st derivative equals the slope of *P(x)* at each position *x*. With the slope we could define the five pixel bands corresponding to the defined depth parallel to the cortex edge. Figure 1 visualizes the mode of pixel band selection. We will refer to the bands throughout the text as 1st to 5th pixel band.

We calculated the following three parameters for each of the five pixel bands:

- The maximum intensity *I*_*max*_, we observed in the pixel band in the 50 frames (110 ms) after thalamic stimulation, e.g., for the 1st band: *I*_*max,1st*_
- The duration *T*_*max*_ from stimulus to *I*_*max*_, e.g., for the 1st band: *T*_*max,1st*_
- The proportion of pixels *PP*_*framemax*_ in the band that showed a temporal maximum intensity in at least one of the 50 frames, e.g., for the 1st band: *PP*_*framemax,1st*._ Therefore we detected the pixel location of the intensity maximum for each frame.

These three parameters help to address following questions:

- Changes in *I*_*max*_: Does the anesthetic dampen or intensify the cortical response to thalamic stimulation?
- Changes in *T*_*max*_: Does the anesthetic delay or accelerate the cortical response to thalamic stimulation?
- Changes in *PP*_*framemax*_: Does the anesthetic change the stimulus propagation pathway among cortical neurons?

The relative changes we show in results present the ratio of control/substance for each experiment and each layer. This means, we evaluated the individual changes.

### Statistical analysis

For the evaluation of the general cortical response we used MATLAB® to perform a Kruskall-Wallis test, together with a post hoc Tukey-Kramer test to check for concentration dependent differences. The corrected level for significance was *p* < 0.05. We used Hedges' g for dependent data from the MATLAB® based MES tool box (Hentschke and Stuttgen, 2011) as measure of effect size to evaluate anesthetic-induced changes in the overall cortical response as well as the response in the pixel bands to a thalamic stimulus. In addition to the *g*-value, we estimated the 95% confidence intervals (CI) with a 10,000-fold bootstrapping. A 95% CI exclusive zero indicates significance (*p* < 0.05).

## Results

### Cortical depolarization upon thalamic stimulation

**Figure 2:**
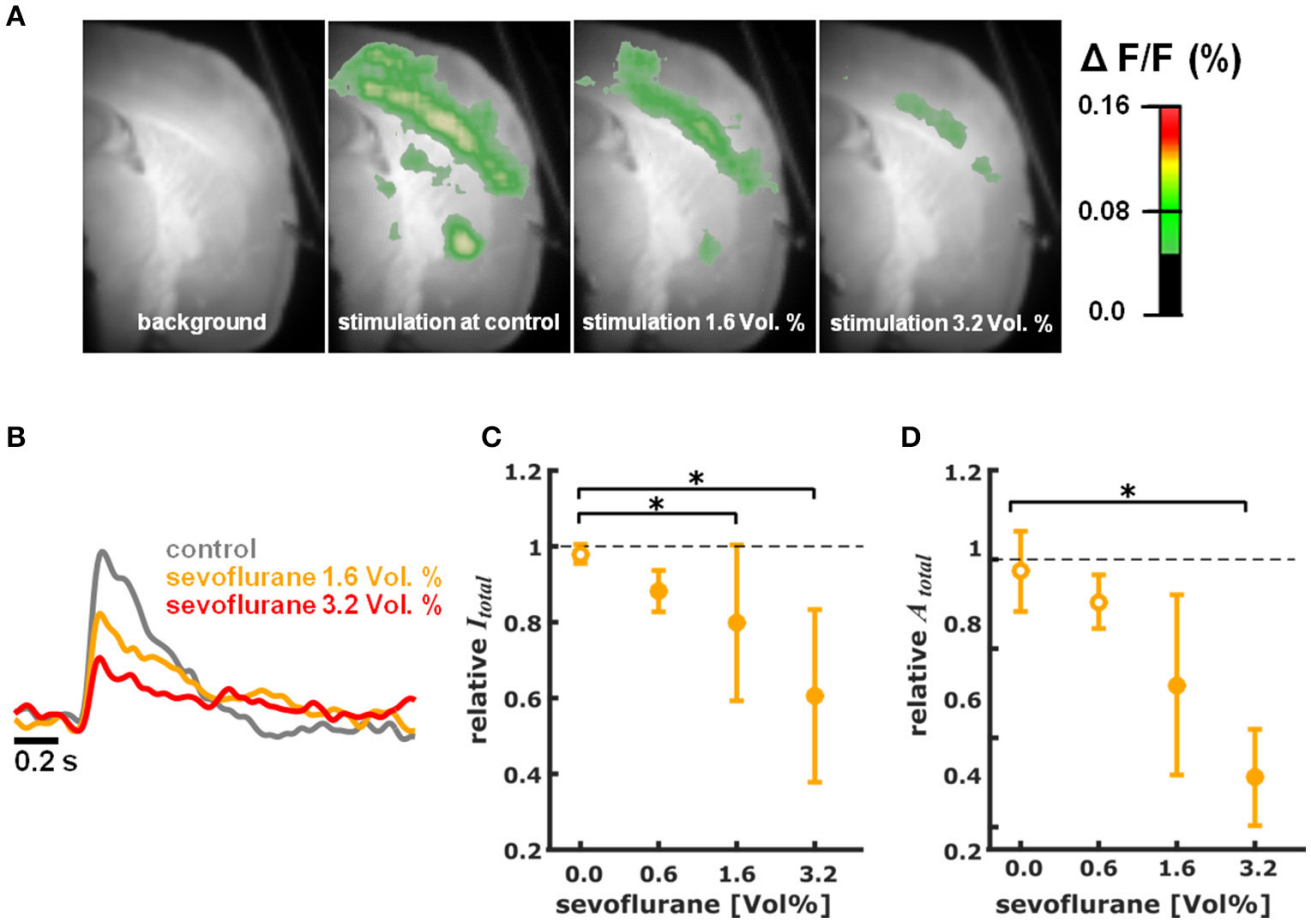
Sevoflurane dose-dependently reduces intensity and area of cortical depolarization upon thalamic stimulation. **(A)** Representative pictures showing fluorescence intensity of an experiment with thalamic stimulation in the absence (control) and presence of different concentrations of sevoflurane. Time of recording of these pictures was 22 ms after thalamic stimulation. **(B)** Representative fast depolarization-mediated signal traces in the absence and presence of sevoflurane. **(C)** Sevoflurane decreased the intensity of cortical depolarization (amplitude of depolarization-mediated fluorescence signals; relative to control presented as median ± median absolute deviation) in a concentration dependent manner. The Kruskall-Wallis test led to a *p* = 0.006 and the post-hoc test showed significant differences between control conditions and the 1.6 and 3.2% sevoflurane concentrations. The Hedges' g indicated strong effects at all concentrations. **(D)** Sevoflurane decreased the area of cortical depolarization to (relative to control presented as median ± median absolute deviation). The Kruskall-Wallis test led to a *p* = 0.006 and the post-hoc test showed significant differences between control conditions and the 3.2% sevoflurane concentration. The Hedges' g indicated strong effects for 1.6 and 3.2% sevoflurane. ^*^ indicates significant differences derived from the *post-hoc* analysis. Filled dots indicate a significant differences (Hedges' g; 95% confidence interval exclusive 0) when compared to control conditions. Sample size was *n* = 5 at each level.

**Figure 3:**
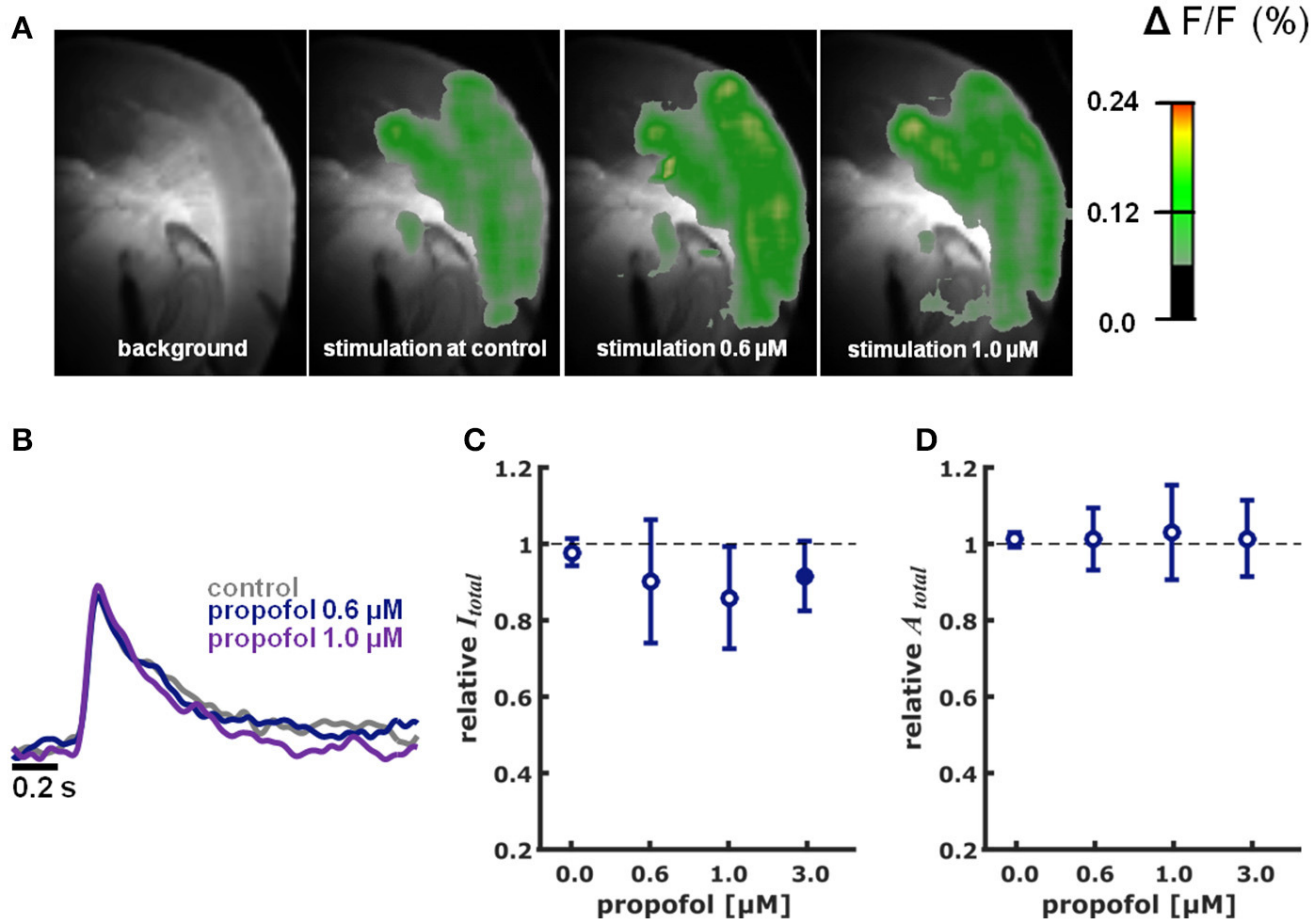
Propofol in different concentrations did neither impact intensity nor area of cortical depolarization upon thalamic stimulation. **(A)** Representative pictures showing fluorescence intensity of an experiment with thalamic stimulation in the absence (control) and presence of different concentrations of propofol. Time of recording of these pictures was 22 ms after thalamic stimulation. **(B)** Representative fast depolarization-mediated signal traces in the absence and presence of propofol. **(C)** Propofol did not affect the intensity of cortical depolarization (amplitude of depolarization-mediated fluorescence signals; relative to control as median ± median absolute deviation). The Kruskall-Wallis test led to a *p* = 0.673, not neglecting the null hypothesis of identical distributions. Hedges' g revealed a strong decreasing effect of propofol on intensity at the 3.0 μM concentration level. **(D)** Propofol did not affect the area of cortical depolarization to (relative to control presented as median ± median absolute deviation): The Kruskall-Wallis test led to a *p* = 0.997, not neglecting the null hypothesis of identical distributions. Hedges' g revealed no effect of propofol on the area of cortical depolarization. ^*^ Indicates significant difference derived from the *post-hoc* analysis. Filled dots indicate a significant differences (Hedges' g; 95% confidence interval exclusive 0) when compared to control conditions. Sample size was *n* = 5–6 at each level.

Figure S1 illustrates the cortical depolarization upon electrical stimulation of the ventrobasal thalamus under control conditions. Figures 2A,B, 3A,B are representative VSDI recordings and fluorescent intensity curves for sevoflurane and propofol, respectively.

#### Sevoflurane

Sevoflurane dose-dependently and significantly (*p* = 0.006, Kruskal-Wallis) reduced the intensity of cortical depolarization (*I*_*total*_) upon thalamic stimulation. The relative *I*_*total*_ decreased to 0.88 (0.05) median (mad) at 0.6%, to 0.80 (0.21) at 1.6%, and to 0.61 (0.23) at 3.2% sevoflurane. Hedges' g indicated a strong decrease in intensity at each concentration level compared to baseline (Figure 2C). Hedges' g (and 95% CI) were 1.56 [1.21 7.54] for 0.6% sevoflurane; 1.28 [0.93 4.55] for 1.6% sevoflurane; and 1.90 [1.03 4.99] for 3.2% sevoflurane. Analogously, sevoflurane attenuated the area (A_*total*_) of cortical depolarization following electric stimulation of the thalamus in a dose-dependent manner (*p* = 0.006, Kruskal-Wallis, Figure 2D). The relative A_*total*_ was 0.91 (0.06) median (mad) at 0.6%, to 0.72 (0.20) at 1.6%, and to 0.51 (0.11) at 3.2% sevoflurane. Post-hoc analysis revealed a significant difference of 3.2% sevoflurane compared to control conditions. According to the Hedges' g, the attenuation was strong and significant except for the 0.6% concentration level (Figures 2C,D). Hedges' g was 0.61 [−0.15 2.31] for 0.6% sevoflurane; 1.37 [0.61 4.08] for 1.6% sevoflurane; and 2.91 [2.41 6.46] for 3.2% sevoflurane, when compared versus control conditions.

#### Propofol

Propofol did neither change *I*_*total*_ (*p* = 0.673, Kruskal-Wallis) nor *A*_*total*_ (*p* = 0.997, Kruskal-Wallis) in a concentration-dependent fashion (Figures 3A–D). The relative changes in *I*_*total*_ were: 0.6 μM propofol: 0.94 (0.16); propofol 1 μM: 0.86 (0.13); propofol 3.0 μM 0.92 (0.09) (*n* = 5 for each data point). Quantification of the effect size relative to control using Hedges' g revealed decreasing effect of propofol on *I*_*total*_ at the 3.0 μM concentration level. Hedges' g (and 95% CI) were 0.41 [−0.62 8.49] for 0.6 μM propofol; 0.60 [−0.22 6.45] for 1.0 μM propofol; and 0.99 [0.21 2.23] for 3.0 μM propofol. Compared to control conditions, relative *A*_*total*_ was 1.01 (0.08) at 0.6 μM propofol; 1.03 (0.12) at 1.0 μM propofol and 1.02 (0.10) at 3.0 μM propofol. The Hedges' g analysis revealed no effect of 0.6 μM propofol (0.10 [−1.13 1.28]), 1.0 μM propofol (0.26 [−1.27 1.43]), and 3.0 μM propofol (−0.02 [−0.91 1.43]) on *A*_*total*_.

### Response in cortical pixel bands

The analysis of the pixel bands revealed that 3.2% sevoflurane significantly reduced *I*_*max*_ in each of the five bands. Sevoflurane further caused a significant delay of cortical response to thalamic stimulation, i.e., an increase of *T*_*max*_ in the 1st and 5th pixel bands. Time to max increased from 31 ms (mean) to 52 ms in the 5th pixel band and from 35 ms to 54 ms in the 1st pixel band. Stimulus propagation inclusive averaged durations were: 4th: 30 ms -> 5th: 31 ms -> 1st: 35 ms -> 3rd: 36 ms -> 2nd: 39 ms at control and: 2nd: 42 ms -> 3rd: 43 ms -> 4th: 45 ms -> 5th: 52 ms -> 1st: 54 ms at 3.2% sevoflurane.

**Figure 4:**
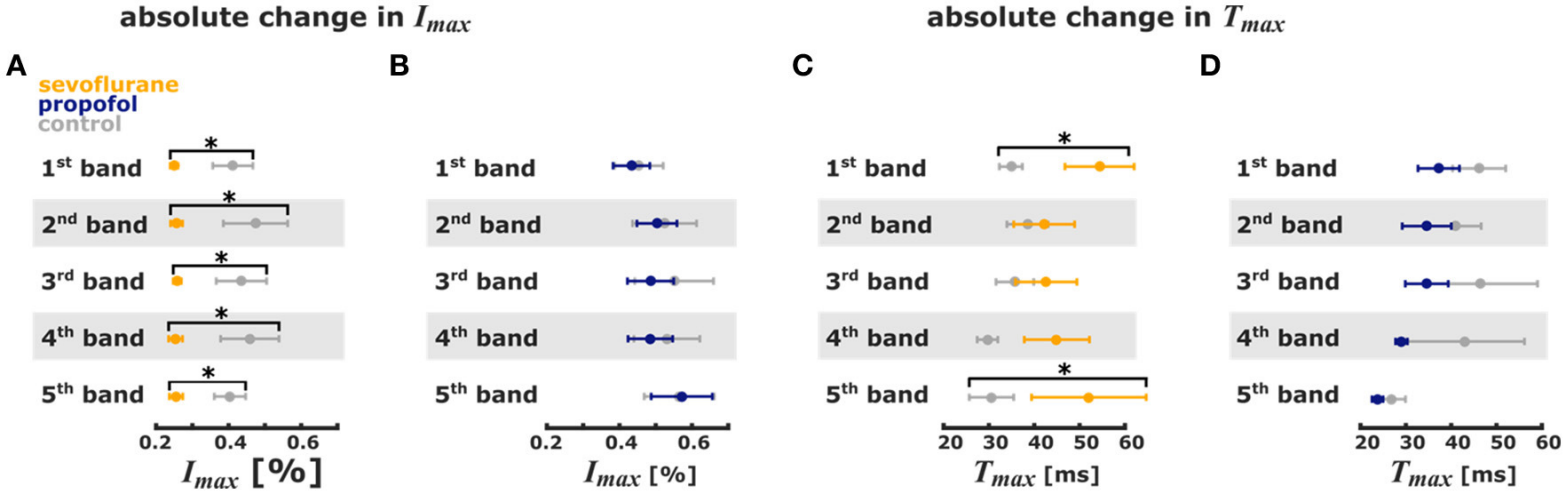
Sevoflurane and propofol affect depolarization of cortical layers in a substance-specific manner. Error bars (mean ± sem, *n* = 5) of absolute stimulus propagation times and absolute maximum changes in fluorescence. **(A)** 3.2% sevoflurane significantly reduced the maximum change in fluorescence activity after the stimulus in all 5 pixel bands. **(B)** 1 μm propofol had no effect on the stimulus-induced change in fluorescence activity. **(C)** 3.2% sevoflurane significantly prolonged the time to maximum change in fluorescence intensity in the 1st and 5th pixel band. **(D)** 1 μm propofol had no effect the time to maximum change in fluorescence intensity. ^*^ indicates significant differences derived from the Hedges' g test (95% confidence interval exclusive 0).

Further, specificity of stimulus propagation decreased as evident by significantly higher *PP*_*framemax*_ in the 1st and 5th pixel bands. With 1 μM propofol, we did not observe any significant changes in the timing of cortical response, suggesting that propagation of intracortical communication was unaffected by propofol at these concentrations. The stimulus propagation was: 5th: 27 ms -> 2nd: 41 ms -> 4th: 43 ms -> 1st: 46 ms ->3rd: 46 ms at control and: 5th: 24 ms -> 4th: 29 ms -> 3rd/4th: 35 ms -> 5th: 37 ms at 1.0 μM propofol. Figure 4B contains the detailed *T*_*max*_ information. Figures 4A,B contains the detailed Imax information and Figures 4C,D contains the detailed *T*_*max*_ information.

**Figure 5:**
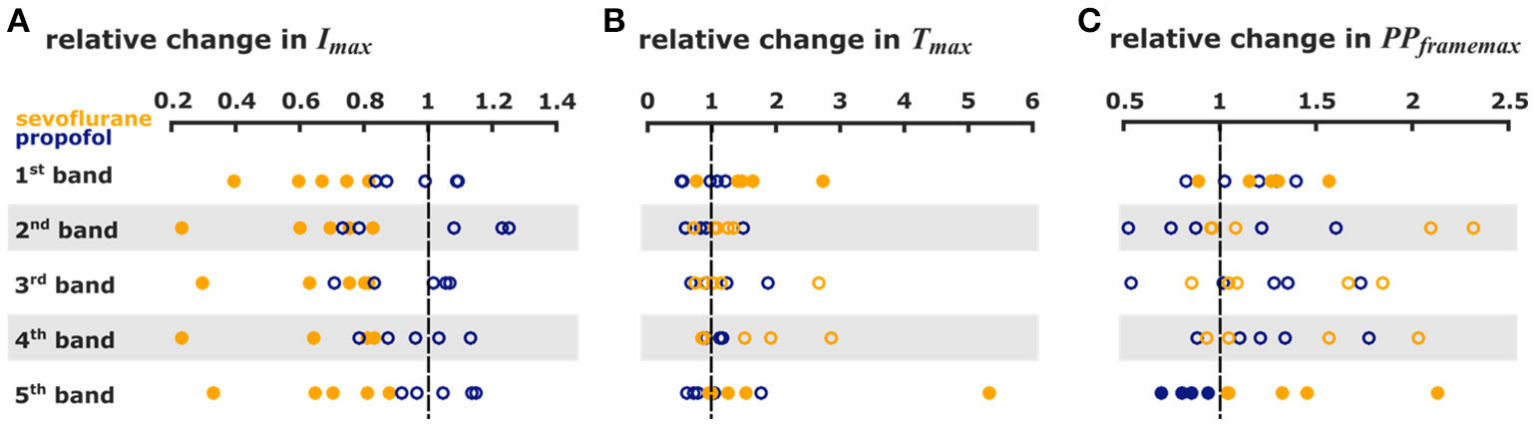
Sevoflurane and propofol affect depolarization of cortical layers in a substance-specific manner. Scatter plots of the relative change in the single pixel band caused by sevoflurane (orange) and propofol (blue). Filled dots indicate a significant change, i.e., 95% CI excluding zero. **(A)** The maximum intensities in the 110 ms after stimulus were significantly lower with sevoflurane in all pixel bands. **(B)** The time to the absolute maximum was significantly increased with sevoflurane in the 1st and 5th pixel band. **(C)** Sevoflurane also caused a significant increase in maximum distribution in the 1st and 5th pixel band, while propofol caused a significant decrease in the maximum distribution 5th pixel band. The detailed results of the Hedges' g analysis are presented in Table 1.

But we observed a significant decrease of *PP*_*framemax*_ in the 5th pixel band. Figures 5A–C presents the single parameter values. Table 1 contains the results from the Hedges' g analysis indicating strength and significance of a possible effect.

**Table 1:** Results of the Hedges' g analysis for the sevoflurane- and propofol-indiced changes of intensity *I*_*max*_, time delay *T*_*max*_, and maximum distribution *PP*_*framemax*_ for the 1st−5th single pixel band.

**Pixel band**	**Relative Change *I_max_***		**Relative Change *T_max_***		**Relative Change *PP_framemax_***	
	**Sevoflurane**	**Propofol**	**Sevoflurane**	**Propofol**	**Sevoflurane**	**Propofol**
1st	**1.47 [1.28 6.58]**	**0.01 [**−**0.89 0.63]**	−1.24 [−4.79 −0.23]	0.61 [−0.33 2.18]	−**0.70** −**4.43** −**0.10]**	−0.22 [−0.75 0.12]
2nd	**1.23 [1.09 5.40]**	0.12 [−0.31 0.46]	−0.23 [−0.80 0.30]	0.41 [−0.58 2.19]	−0.99 [−2.16 0.15]	0.03 [−0.79 2.12]
3rd	**1.29 [1.14 4.68]**	0.26 [−0.13 0.75]	−0.44 [−1.77 0.49]	0.45 [−1.12 1.63]	−0.79 [−1.81 0.33]	−0.20 [−0.97 0.55]
4th	**1.27 [0.09 4.56]**	0.21 [−0.09 0.95]	−1.04 [−3.23 0.11]	0.54 [−1.07 1.41]	−0.81 [−1.91 1.52]	−0.36 [−1.40 0.04]
5th	**1.62 [1.26 3.58]**	−**0.03 [**−**0.20 0.39]**	−0.81 [−2.00−0.04]	0.49 [−0.90 4.79]	−**1.20 [**−**2.86** −**0.53]**	**0.22 [0.01 1.00]**

*Bold cells indicate a significant effect*.

## Discussion

We evaluated the effect of sevoflurane and propofol on cortical depolarization following an electric stimulus applied to the thalamic VPM. While we observed a sevoflurane-induced reduction in intensity and activated cortical area following the stimulus, we found that propofol did not change these parameters. The analysis of defined cortical pixel bands revealed that propofol only affected the distribution of intensity maxima in the 5th pixel band, i.e., propofol seems to specifically constrict the stimulus propagation in this band. Sevoflurane in contrast acted on a wide variety of parameters. It reduced the maximum intensity in all five pixel bands and delayed the cortical response to thalamic stimulation. Interestingly, the number of pixels that contained a maximum in a frame increased in the 1st and 5th pixel bands (i.e., in the upper and lower cortical layers) with sevoflurane exposure, perhaps reflecting less specificity in the effects of sevoflurane on cortical information processing. When looking at the absolute stimulus propagation times, we found the first intensity maxima following the thalamic stimulus around 20–30 ms after the stimulus in the 4th and 5th pixel bands. Hence, these bands seem to reflect the input into the cortical area in the deep cortical layers. These findings underline that sevoflurane and propofol seem to induce general anesthesia in different ways as might be expected from drugs of different molecular families. Propofol mainly acts via GABA_A_ receptors, whereas sevoflurane in clinically relevant concentration enhances GABA signaling (Garcia et al., 2010) but additionally affects glycine and glutamate receptors (Rudolph and Antkowiak, 2004; Grasshoff et al., 2006).

The minimum alveolar concentration (MAC) defines the anesthetic potency of inhalational anesthetics in terms of a motor response upon a painful stimulus (Eger et al., 1965). MAC values vary among species, and in the case of the species *mouse*, among strains (Sonner et al., 2000). For mice, a MAC value of 3.22 vol.% for sevoflurane (Ichinose et al., 1998) has been published. We applied sevoflurane by passing the carbogen gas through a calibrated vaporizer. Concentrations given reflect dial settings of the vaporizer. The fact that we did not measure the aqueous concentration (c_aq_) of sevoflurane might be a limitation of our study. In our experiments sevoflurane reduced the cortical response upon thalamic stimulation with a calculated IC_50_ of 3.18%. When calculated with published solubility coefficients for sevoflurane in aqueous solution (Franks and Lieb, 1993, 1996), the application of 1 MAC (3.2%) sevoflurane at room temperature would lead to a calculated c_aq_ of 0.38 mM. A previous study reported a c_aq_ of 0.23 mM by application of 2.8% sevoflurane. Other work reported a linear solubility of sevoflurane in aCSF with application of 2.0% sevoflurane leading to a c_aq_ of 0.42 mM (Haseneder et al., 2009; Nishikawa et al., 2011). Thus, we expect the c_aq_ of sevoflurane after application of 3.2% sevoflurane under our experimental conditions to be between 0.26 and 0.67 mM. Hence, the applied concentrations of sevoflurane lead to aqueous concentrations that are well in a clinical relevant range and therefore the attenuation of intensity and area of cortical depolarization by sevoflurane occurred at clinically relevant concentrations.

In brain slice preparations, propofol slowly equilibrates in brain tissue and the equilibration time depends on the penetration depth. The recordings in our study were performed after an equilibration time of 30 min. This time is in accordance with patch-clamp studies in acute brain slices that report propofol-induced neuropharmacologic effects at comparable aqueous concentrations of propofol (0.3 μM; Ying and Goldstein, 2005a, 0.6 μM; Ying and Goldstein, 2005b, and 5 μM Chen et al., 2005; Ying et al., 2006) after an equilibration time of 5–20 min. Patch-clamp recordings in acute brain slices are usually obtained from superficially located neurons. Analogously, the recorded VSDI signals have been shown to originate from superficial neuronal structures located up to 50–75 μm deep in the thalamocortical brain slice, since the dye Di-4-ANNEPPS seems to penetrate that deep (Hill and Greenfield, 2013). Published data suggests that at a depth of 50 μm, 50% of the final equilibrium concentration is reached 30 min after propofol application (Gredell et al., 2004). Hence experiments performed at distinct propofol concentrations might underestimate the propofol-induced effects in the brain if unequilibrated. A propofol blood concentration of 3.5 μg/ml seems to produce loss of righting reflex in rodents. This corresponds to a free, i.e., not protein bound, concentration of 0.2 μM (Gredell et al., 2004). We applied propofol in concentrations up to 3 μM. Therefore, our experiments were performed at clinically relevant concentrations despite the slow diffusion kinetics of propofol. A failure to investigate the acute and dynamic effects of propofol on thalamocortical slice physiology is a limitation of our chosen technique. Besides the different actions of sevoflurane and propofol on molecular targets, these differences can be observed at a larger scale.

Phenotypically, the loss of consciousness induced by sevoflurane or propofol seems similar. Human EEG studies revealed a loss of cortical feedback connectivity as a key mechanism of sevoflurane or propofol induced unconsciousness (Ku et al., 2011; Jordan et al., 2013; Ranft et al., 2016). But during general anesthesia patients develop different spectral (frontal) EEG patterns for sevoflurane and propofol (Akeju et al., 2014). The sevoflurane spectrogram shows an unspecific activation of low EEG frequencies especially in the theta range, while propofol causes a distinct alpha peak. Further, the EEG burst suppression pattern in rats, a signature for very deep anesthesia, appears different for sevoflurane and propofol (Kenny et al., 2014); reinforcing the notion that cortical influences of these two drugs differ.

EEG recordings mainly reflect cortical activity that can be heavily influenced by subcortical structures. Electrophysiological and optical (VSDI) signals result from changes in membrane potential, i.e., from transmembrane voltage changes (Buzsáki et al., 2012). Contreras et al could show a similarity of electrophysiological (LFP) and optical (VSDI) evoked responses (Contreras et al., 2017). They also showed a similarity between these signals for 1 s episodes of spontaneous activity. And since LFP activity can be considered “micro-EEG” (Buzsáki et al., 2012), i.e., the EEG being a more generalized and more blurred version of an LFP covering a larger part of the cortical network, we are confident that findings from electrophysiological and optical (VSDI) experiment can be compared to a big degree. The use of fMRI demonstrates that anesthetics also affect subcortical structures such as the thalamus (Mhuircheartaigh et al., 2010). The investigation of anesthetic-induced effects on interactions between cortical and thalamic areas is highly relevant, since they are necessary to maintain consciousness (Mashour and Alkire, 2013). Distortion of these interactions seems a general key mechanism of anesthesia induced unconsciousness (John and Prichep, 2005).

Latest results from fMRI suggest a difference in how the anesthetics, sevoflurane or propofol, affect the connectivity between thalamus and cortex. Sevoflurane did not cause a significant change in the connectivity between thalamus and sensory cortices (Ranft et al., 2016). Propofol in contrast evoked an uncoupling of the thalamus and cortical independent component networks (Jordan et al., 2013). Of course, the findings from the MRI and VSDI experiments cannot be directly related, but the similarity of the findings highlights the differences between sevoflurane and propofol, when it comes to thalamocortical effects.

Our results revealed that propofol may have a more specific mode of action to negatively influence network activity between thalamus and cortex than sevoflurane. While sevoflurane affected the cortex in general, i.e., in all five observed pixel bands, we only found a propofol effect in the 5th band. Our findings of a persistent thalamocortical stimulus propagation structure with sevoflurane and a funneling of this propagation in the 5th with propofol seem to be in line with the mentioned observations from functional fMRI. One limitation in the VSDI recordings is a somewhat coarse spatial resolution, the fit calculation that transfers the selection into an arc line, and the pixel size. It is therefore not possible to draw definitive conclusions from the pixel bands to effects that are specific for distinct cortical layers. Previous work (Cruikshank et al., 2010) showed, that axonal arbors from thalamocortical projections are concentrated in two regions that are localized further than 500 μm from the edge of the brain in the same angled slice preparation we used for our experiments. Since thalamocortical neurons primarily send projections to cortical layers IV and VI (Lee and Imaizumi, 2013), our results from the 5th band correspond to the deep cortical layers, most likely layer IV–VI.

Cortical layer IV plays a special role in thalamocortical processing (Berbel et al., 2010; Christianson et al., 2011); it serves as input of feed-forward excitation from thalamic core cells, neurons from VPL and VPM (Jones, 1998). Thalamic information undergoes initial intracortical signal processing in cortical layer IV. From there, information is relayed to supragranular laminae, i.e., cortical laver I and II (Jones, 1998). An anesthetic-induced modulation of neurons in layer IV might therefore result in impaired cortical propagation of signals initiated in the thalamus. Cortical layer VI contains pyramidal neurons that provide excitatory feedback to the thalamus (Alitto and Usrey, 2003). Together with cortical layer IV, it presents an important part of the communication structure between thalamus and cortex.

Under the influence of propofol we saw a reduced distribution of depolarization in these deep cortical layers following electric stimulation of the thalamus. Changes in dye fluorescence of each pixel represent the combined neuronal activity of axons, dendrites, neuronal cell bodies and glia. Because the surface area of dendrites is much larger than that of somata or axons, the origin of the recorded signal is mainly dendritic (Yuste et al., 1997). In accordance with other authors (Hill and Greenfield, 2013) we conclude that our recordings reflect the activity in the postsynaptic arborization of the thalamocortical afferents. In the cortex simple sensory stimuli lead to concomitant occurrence of synaptic excitation and inhibition (Isaacson and Scanziani, 2011). Principal and inhibitory neurons are reciprocally connected and local network activity is consequently modulated by inhibitory feedback. As a GABA_A_ receptor agonist, propofol enhances inhibitory synaptic transmission (Eckle et al., 2015) and consequently shifts the balance of excitation and inhibition toward inhibition. Hence, reduced spatial distribution of pixels presenting maximum intensity over a period of time (110 ms) is highly likely to originate from augmented local recurrent inhibition of the deep cortical structures involved in communication between thalamus and cortex. This inhibition and the funneling effect may lead or contribute to decoupling between thalamus and cortex. However, we cannot draw a definitive conclusion from our experimental data, especially if afferent or efferent cortical connections to thalamus or both are affected.

In contrast, sevoflurane caused a general decrease in depolarization upon thalamic stimulation in all cortical layers. In contrast to propofol, sevoflurane impairs local field potential activity in organotypic slice cultures of isolated cortex (Drexler et al., 2013). These findings agree with our results of sevoflurane affecting our parameters in all cortical layers, whereas propofol only affects one parameter in the 5th pixel band.

In addition to the potentiation of GABAergic inhibition, sevoflurane enhances glycine receptor activity and impairs excitatory synaptic transmission at clinical relevant concentrations (Rudolph and Antkowiak, 2004). Hence, the sevoflurane-induced general attenuation of cortical depolarization that we observed in our study possibly results either from reduced excitatory feed-forward inputs from the thalamus or impaired corticocortical neurotransmission. Contrary to propofol, sevoflurane increased the spatial distribution of intensity maxima in the 5th pixel band. It is possible that the effect of sevoflurane on excitatory pathways may be more important for producing unconsciousness than its effect on enhancing activity of local inhibitory networks. This is in line with findings showing that volatile anesthetics mainly act on glutamine-mediated orthodromic pathways in the hippocampus whereas propofol enhances recurrent inhibition (Asahi et al., 2006).

In agreement with our data, Raz et al. detected a strong effect of another volatile anesthetic, isoflurane, on corticocortical processing in the auditory and visual cortex (Raz et al., 2014). The authors describe that isoflurane preferentially inhibits “top-down” projections. With our approach of thalamic stimulation, we could observe significant effects of sevoflurane on the “bottom-up” thalamocortical pathway. These findings may seem contradictory, but the authors state that the action of isoflurane is pathway specific. In contrast to Raz and co-workers we applied electric stimuli to a thalamic nucleus mainly responsible for relaying sensory information (Miyata, 2007) in our experiments. Hence, the results may not be comparable.

Liu et al. (2013) further found that propofol also affects the thalamocortical system. The ventrobasal complex of the thalamus is a first order relay (specific system) because it transmits information from a subcortical source (lemniscal projections; Andersen et al., 1964; Sherman and Guillery, 1996) directly to the cortex. We placed the stimulation electrode onto the ventrobasal complex for our voltage-sensitive dye imaging experiments. Higher-order pathways seem to be preserved in the thalamocortical slice preparation (Lee and Sherman, 2008). We cannot completely exclude that our electrical pulse applied to the ventrobasal complex also stimulated higher-order nuclei of the thalamus finally resulting in cortical depolarization. This might limit the reliability of our findings with respect to their specificity to the ventrobasal complex and we cannot conclude whether propofol also affects the nonspecific system.

In summary, we have demonstrated that sevoflurane and propofol affect thalamocortical stimulus propagation in a substance-specific manner. Sevoflurane globally decreased cortical depolarization upon thalamic stimulation whereas propofol specifically attenuated signal processing in the deep cortical areas. This provides further evidence that the mechanisms of anesthetic-induced loss of consciousness are drug- and pathway-specific.

## Author contributions

SK designed and performed experiments, wrote manuscript; CM performed experiments and provided critical feedback on manuscript; SS, EK, PG, GS, and GR provided critical feedback on the manuscript; RH designed experiments and provided critical feedback on manuscript; MK designed and conducted analysis, wrote manuscript.

### Conflict of interest statement

The authors declare that the research was conducted in the absence of any commercial or financial relationships that could be construed as a potential conflict of interest.

## Supplementary material

Figure S1 Cortical response after an electrical thalamic stimulation over 41.8 ms at baseline condition. The figure shows the VSDI response in the cortex after electrical stimulation of the ventrobasal nucleus of the thalamus. At ~4.4 ms the stimulus-induced change in fluorescence in the thalamus is visible. The first response in cortical areas develops after ~8.8 ms. After ~29–31 ms the cortical response seems to start to fade.
