# Supplementary figures and images for: Propofol and Sevoflurane Differentially Modulate Cortical Depolarization following Electric Stimulation of the Ventrobasal Thalamus
Source: Front Comput Neurosci. 2017 Dec 11;11:109. doi: 10.3389/fncom.2017.00109 (PMC5732174; doi:10.3389/fncom.2017.00109)

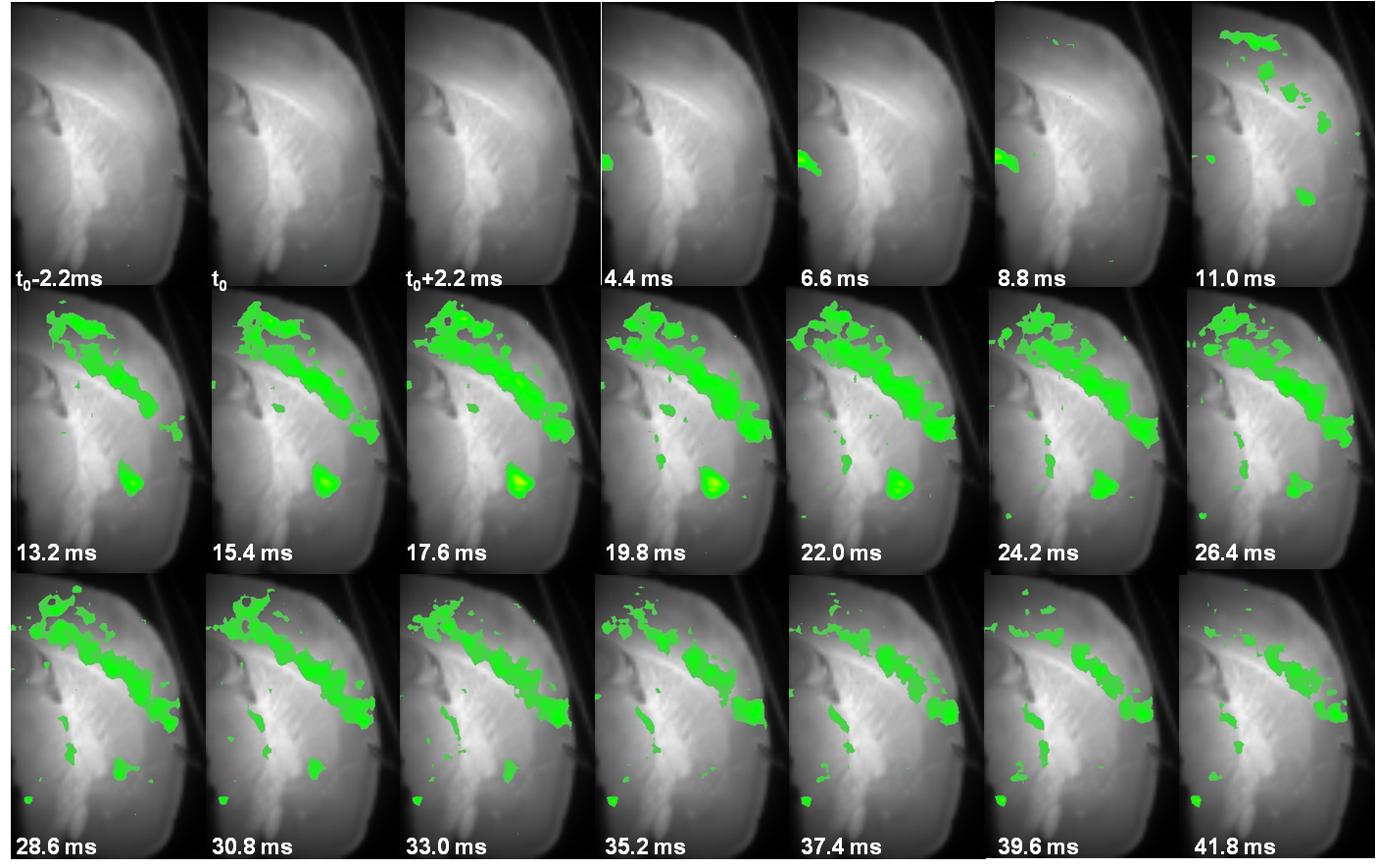

Supplement: Figure S1 — Cortical response after an electrical thalamic stimulation over 41.8 ms at baseline condition. The figure shows the VSDI response in the cortex after electrical stimulation of the ventrobasal nucleus of the thalamus. At ~4.4 ms the stimulus-induced change in fluorescence in the thalamus is visible. The first response in cortical areas develops after ~8.8 ms. After ~29–31 ms the cortical response seems to start to fade. [file Image1.TIF]
